# A psychological effect of having a potentially viable sequestration strategy

**DOI:** 10.1186/1750-0680-1-4

**Published:** 2006-07-13

**Authors:** Katsumi Matsumoto

**Affiliations:** 1 Department of Geology and Geophysics, University of Minnesota, Minneapolis, MN, 55455, USA

## Abstract

Purposeful carbon sequestration by direct injection into the deep ocean can store carbon for centuries. Even after injected carbon begins to leak back out to the atmosphere, much of the injected carbon will remain sequestered because of the acid neutralizing capacity of seawater. The slow leakage that occurs centuries into the future can give a false sense of security that the carbon and climate problem is under control. If this were to cause policy makers to become less vigilant about reducing the total emissions of anthropogenic carbon, our descendants would be penalized with having much higher carbon dioxide content in the atmosphere when leakage begins. This "carelessness feedback" would apply to other forms of sequestration that are not permanent. To avoid falling into this trap requires generations of policy makers to be aware of the feedback and committed to intergenerational equity.

## Background

It is now widely recognized that the carbon dioxide (CO_2_) we emit to the atmosphere is the dominant agent of climate change in the Anthropocene [1]. There is mounting pressure to curb emissions of this greenhouse gas from various segments of the global society, including advocates of environmental protection and sustainability, and citizens concerned with equity between counties and generations. A culmination of this movement is the 2005 entry into force of the Kyoto Protocol. With the United States unwilling to ratify the protocol, it was essentially Russia's ratification that satisfied the 55% clause and allowed the protocol to enter into force. However, the realization that meaningful reduction in emissions is very difficult to achieve even with the Kyoto Protocol has prompted many countries to pursue geoengineering strategies to purposefully sequester carbon both on land and in the oceans. Research and promotion of purposeful sequestration are active. For example, in the US, there is an annual meeting devoted to this subject that is supported in part by the federal government (see ).

There is potential danger in pursuing purposeful sequestration however, because it can give a sense of security that the solution to CO_2 _emissions problem is at hand. This sense of security may well erode what little political will there is to curb emissions. After all, if there is going to be an effective and long term means to store CO_2_, why should we even bother reducing fossil fuel consumption in the first place? Is it not just economically painful and politically unpopular? The real danger is if this sense of security is unfounded.

Here I discuss this danger in the context of direct injection of carbon into the deep ocean, a form of sequestration that involves leakage in the future. Implications will be relevant for all other forms of sequestration that also are not permanent.

## Discussion

A primary reason why the oceans may be an attractive reservoir to purposefully sequester carbon is that they have a very large capacity to hold and absorb carbon due to the alkaline chemistry of seawater. This large capacity is evident from the fact that the oceans have about sixty times more inorganic carbon than the atmosphere. For this reason, a large fraction (but not all) of the CO_2 _that is emitted to the atmosphere will be taken up ultimately by the oceans. If the CO_2 _neutralizing capacity of calcium carbonate in ocean sediments is included, the capacity is even larger.

Today anthropogenic CO_2 _is accumulating in the atmosphere despite this, because the transfer of CO_2 _from the atmosphere to the oceans is rather slow. Roughly it takes about one year for the surface ocean to come into equilibrium with the overlying atmosphere with respect to CO_2_. One can think of this as the time it takes for the surface ocean to become saturated with CO_2_. The more serious time limiting step is the ventilation of the ocean interior. The time it takes to mix the CO_2_-saturated surface waters into the upper ocean (say the top 1000 m above the thermocline) and store CO_2 _there is decades. It takes centuries to ventilate the vast deep ocean.

Purposeful sequestration of anthropogenic carbon into the deep ocean by direct injection, as first proposed in 1977 by Marchetti [2], would bypass these slow transfer processes. Once sequestered, the long ventilation time assures that CO_2 _will remain in the deep ocean for centuries. The high sequestration efficiency has been confirmed in a number of modeling studies, as noted in a recent IPCC special report on CO_2 _capture and storage[3]. However, ocean sequestration involves leakage, whereby some fraction of the injected CO_2 _will escape to the atmosphere. This occurs because the injected CO_2 _may reach the surface ocean without being sufficiently diluted by oceanic mixing. More importantly, it occurs because, as noted above, not all of the CO_2 _emitted will be absorbed ultimately by the oceans. The final, steady state fractions that will reside in the atmosphere and the oceans do not in fact depend on whether CO_2 _is initially injected into the oceans or emitted to the atmosphere [4]. They are determined solely by the accumulated carbon perturbation (i.e., total CO_2 _released).

There is potentially serious consequence to the slow, centennial time scale leakage of CO_2_, if we were to *not *curb future emissions as a result of our feeling secure about the effectiveness and permanency of carbon sequestration in the deep ocean. The consequence is that we would be penalized with having much more CO_2 _in the atmosphere than anticipated, because the CO_2 _that we thought we had gotten rid of by sequestration would come leaking back out of the deep ocean. Christoph Heinze calls this the "carelessness feedback" (personal communication). This is somewhat analogous to a risk that already has been identified, namely that CO_2 _emissions in the future may increase, if today's carbon conserving practices are abandoned [5]. For example, discontinuing fire control in forests (analogous to purposeful sequestration) may result in a rapid loss of at least part of the carbon accumulated during previous years (analogous to leakage).

## Conclusion

The danger of becoming overconfident with purposeful sequestration is greater for those forms of sequestration, such as geologic sequestration, that offer even higher sequestration efficiency and slower rates of leakage. It should be noted that the magnitude of the carbon problem will likely prevent any one technology from solving it completely [6]. So temporary carbon storage strategies will likely be small parts of a broader solution, but the negative effects of leakage coming back to haunt our descendants in the future will not go away. The slow rates of leakage require that succeeding generations of policy makers are cognizant of the "carelessness feedback" and seriously committed to intergenerational equity.

## Competing interests

The author(s) declare that they have no competing interests.

## Authors' contributions

I have written this article in its entirety.

## References

[B1] Crutzen PJ, Stoermer EF (2002). IGBP Newsletter.

[B2] Marchetti C (1977). On geoengineering and the CO2 problem. Climatic Change.

[B3] Metz B, Davidson O, de Coninck HC, Loos M, Meyer LA (2005). IPCC Special Report on Carbon Dioxide Capture and Storage: Prepared by Working Group III of the Intergovernmental Panel on Climate Change.

[B4] Broecker WS, Peng TH (1982). Tracers in the Sea.

[B5] Metz B, Davidson O, Swart R, Pan J (2001). Climate Change 2001 - Mitigation: Contribution of Working Group III to the Third Assessment Report of the Intergovernmental Panel on Climate Change.

[B6] Pacala S, Socolow R (2004). Stabilization wedges: Solving the climate problem for the next 50 years with current technologies. Science.

